# Familial Mediterranean fever in Jordanian Children: single centre experience

**DOI:** 10.31138/mjr.29.4.211

**Published:** 2018-12-18

**Authors:** Raed Alzyoud, Motasem Alsweiti, Hiba Maittah, Ehab Zreqat, Adel Alwahadneh, Mohammed Abu-Shukair, Lana Habahbeh, Mohammed Mutereen

**Affiliations:** 1 Division of Paediatric Immunology, Allergy, and Rheumatology,; 2 Division of Paediatric Gastroenterology, Queen Rania Children’s Hospital,; 3 Department of Pathology, King Hussein Medical Centre, Amman, Jordan

**Keywords:** Familial Mediterranean fever, Jordanian children, Arab Genotypes

## Abstract

**Background:**

Familial Mediterranean fever (FMF) is an autosomal recessive autoinflammatory disorder caused by mutations in the Mediterranean Fever (MEFV) gene. The disease is especially common among Mediterranean ancestry, mostly Armenian, Turkish, Jewish and Arab populations. Our aim is to describe clinical phenotype, and genotype of FMF in the Jordanian children.

**Patients and Methods:**

A retrospective analysis was conducted on paediatric patients who were below 14 years of age and diagnosed as FMF at Queen Rania Children’s Hospital in Jordan between 2014 and 2017.

**Results:**

A total of 196 paediatric patients diagnosed with FMF were included; 54% females and 46% males. The mean age of patients at time of study was 7.8 years, at disease onset was 4.9 years, and at time of diagnosis was 6.6 years. The most common presenting features were abdominal pain (91.8%), fever (73%), arthralgia (16.8 %), and myalgia (12.8%). MEFV gene mutations were homozygous in 47 (24%) patients, heterozygous in 87 (44.4%) patients, compound heterozygous in 55 (28.1%), and negative genotype in 7 (3.6%) patients. Five mutations were the most frequent; M694V, V726A, E148Q, M680I, M694I. All patients were colchicine responsive. We reported only one case of amyloidosis.

**Conclusion:**

The five FMF founder mutations: M694V, V726A, E148Q, M680I, and M694I were the most common in Jordanian children, but had a different order from other ethnic groups.

## INTRODUCTION

Familial Mediterranean fever (FMF) is the most commonly seen autoinflammatory syndrome, and frequently occurs among Turks, Armenians, Jews and Arabs. FMF is an autosomal recessive inherited disease that occurs as a result of point mutation in the Mediterranean Fever (MEFV) gene, on the short arm of chromosome 16 which encodes the protein pyrin.^1^ As FMF is characterized by recurrent episodes of fever and serosal inflammation, the serositis can present as spontaneous attacks of abdominal pain, chest pain, or joint pain, and can also be accompanied with an erysipelas-like skin lesions, acute scrotum, febrile myalgia syndrome, headaches, and/or aseptic meningitis, the attacks resolve within 3–4 days, and the interval between attacks is relatively symptom-free.^2^ The diagnosis of FMF is a clinical one, and molecular analysis of the MEFV gene provides genetic confirmation only. There is a consensus to test for a total of 14 variants: M694V, M694I, M680I, V726A, R761H, A744S, E167D, T267I and I692del are clearly pathogenic, while variants K695R, E148Q, P369S, F479L, and I591T are of unknown significance.^3^ There are different sets of classification and diagnostic criteria for FMF: the first set of criteria was created for adults by the experts in Hashomer Medical Centre in 1997,^2,3^ however, paediatric FMF has some differences in clinical phenotype. Thus, in 2009, a group of paediatric rheumatologists from Turkey who are experts in FMF had designed criteria for children.^4^ Colchicine has been used for more than 40 years in treatment of FMF: its efficacy in reducing the frequency and severity of the episodes, in addition to amyloidosis prevention, had been established.^5^ In this study, we will describe the clinical phenotypes and genotype distribution of FMF in paediatric population at a single centre in Jordan.

## PATIENTS AND METHODS

A retrospective analysis was performed on patients’ records of children who were diagnosed with FMF and were followed at paediatric rheumatology clinic in Queen Rania Children’s Hospital from 2014–2017. All patients were below 14 years of age at time of diagnosis, and followed up for at least six months. FMF diagnosis had been made based on Turkish diagnostic criteria in childhood. Presence of two or more of the following criteria in a patient was accepted as diagnostic criteria for FMF: ≥3 attacks with 6–72 hours duration of fever (axillary temperature of >38° C); abdominal pain; chest pain; oligoarthritis; and positive family history.^4^ All patients were tested for 12 mutations of MEFV gene (E148Q, P369S, F479L, M680I, 1692 DEL, M694V, M694I, M680I, K695R, V726A, A744S, and R761H), using the FMF STRIP ASSAY TM. VIENNA LAB DIAGNOSTICS GmbH.

## RESULTS

Of the total of 196 paediatric patients diagnosed with FMF were being followed at the paediatric rheumatology clinic during the period from January 2014–June 2017, 106 (54%) were females, 90 (46%) were males. All patients were Jordanian; Arab ethnicity. The mean age of patients at time of study was 7.8±3.1 years, mean age at disease onset was 4.9+2.3 years, and mean age at time of diagnosis was 6.6+3.0 years with a mean diagnostic delay of 1.7 years, the youngest patient at time of diagnosis was 6 months old, and the oldest was 14 years old.

**TABLE 1.** Demographic data, clinical features, and associations.

**Age**		
**Mean age at disease onset (± SD)**	4.9 (± 2.3) years	
**Mean age at diagnosis (± SD)**	6.6 (± 3.0) years	
**Mean age at study time (± SD)**	7.8 (± 3.1) years	
**FEATURE**	NUMBER	PERCENT
**Total number**	196	100%
**Males**	90	46%
**Females**	106	54%
**Fever**	180	91.8%
**Abdominal Pain**	143	73.0%
**Family history of FMF**	57	29.1%
**Arthralgia**	33	16.8%
**Myalgia**	25	12.8%
**Chest Pain**	19	9.7%
**Arthritis**	16	8.2%
**Skin Rash**	7	3.6%
**Recurrent HSP**	6	3.1%
**JIA**	6	3.1%
**RAS**	5	2.6%
**Pericarditis**	4	2.0%
**Crohn’s disease**	2	1.0%
**LCV**	1	0.5%
**FSGS**	1	0.5%
**Nephrotic syndrome**	1	0.5%
**Amyloidosis**	1	0.5%
**Mortality**	1	0.5%

SD: standard deviation, HSP: Henoch-Schonlein purpura, JIA: Juvenile Idiopathic Arthritis, LCV: Leukocytoclastic vasculitis, FSGN: Focal segmental glomerulosclerosis, RAS: Recurrent aphthous stomatitis

Clinical features of our cohort are listed in *Table 2*; the most common clinical features were abdominal pain, fever, and musculoskeletal symptoms. Positive family history of FMF was reported in 57 (29.1%) patients. Autoimmune associations accounted for around 10.2%; juvenile idiopathic arthritis in 6 (3.1%) patients, Henoch-Schönlein purpura in 6 (3.1%) patients, recurrent aphthous stomatitis in 5 (2.6%), Crohn’s disease in 2 (1.0%) patients, and systemic leukocytoclastic vasculitis in 1 (0.5%) patient. Renal complications manifested in 3 patients; 1 developed minimal change nephrotic syndrome; 1 had focal segmental glomerulosclerosis and 1 had renal amyloidosis. Mortality rate was only 0.5% (1 patient) who had renal amyloidosis.

**TABLE 2.** MEFV mutations in 196 Jordanian FMF children.

**Mutation**	**Genotype**	**NO.**	**%**
**Heterozygous**	M694V	34	17.3
	E148Q	21	10.7
	V726A	10	5.1
	M680I	10	5.1
	P369S	4	2.0
	A744S	3	1.5
	M694I	3	1.5
	P479L	1	0.5
	F479L	1	0.5
	Total	87	44.4
**Homozygous**	M694V-M694V	32	16.3
	V726A-V726A	6	3.1
	M680I-M680I	7	3.6
	E148Q-E148Q	1	0.5
	M694I-M694I	1	0.5
	Total	47	24.0
**Compound heterozygous**	M694V-V726A	15	7.7
	E148Q-M694V	6	3.1
	M694V-M694I	6	3.1
	E148Q-V726A	6	3.1
	M694I-M680I	6	3.1
	M680I-V726A	4	2
	M680I-M694V	3	1.5
	E148Q-A744S	3	1.5
	M694I-V726A	3	1.5
	R761H-M694V	1	0.5
	M694V-R761H	1	0.5
	M680I-E148Q	1	0.5
	Total	55	28.1
**Negative genotype**		7	3.6

**Figure 1.**
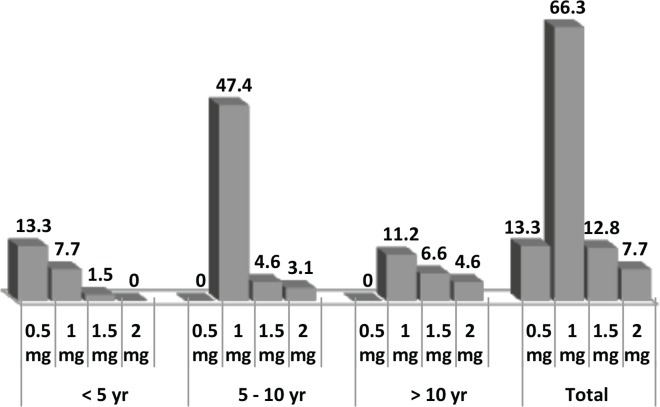
Percentages of colchicine dose in different age groups used in 196 Jordanian FMF children. < 5 yr: less than 5 years old, > 10 yr: more than 10 years old

All patients were tested for 12 mutations of MEFV gene. Patients were classified into 4 categories based on genotype (*Table 3*); homozygous 47 (24%) patients (M694V-M694V was the most common); heterozygous 87 (44.4%) patients (M694V was the most common), compound heterozygous 55 (28.1%); (M694V-V726A was the most common); and no mutation could be identified in 7 (3.6%) patients. Five mutations were the most frequent in the whole cohort (*Table 4*); M694V, V726A, E148Q, M680I, and M694I were detected in 50%, 21.4%, 19.4%, 15.8, and 9.7% respectively either as homozygous, heterozygous or compound heterozygous. All patients were commenced on colchicine since the time of diagnosis, colchicine dosing was: 0.5mg daily in 26 (13.3%) patients; 1mg daily in 130 (66.3%) patients, 1.5mg daily in 25 (12.7%) patients, and 2mg daily in 15 (7.7%) patients, colchicine dose in different age groups in our cohort is summarized in *Figure 1*. All patients showed impressive response on Colchicine; patients were evaluated on monthly bases during the first 6 months to titrate proper colchicine dose, then every 6 months. Both frequency and intensity of FMF attacks reduction at least 50% with normalization of acute phase reactants between attacks were included in response parameters. Colchicine doses were titrated to reach the optimal disease control with the best tolerated dose. Adherence to colchicine treatment was complete in 165 (84.2%) patients, and non-compliant patients were only 31 (15.8%). Colchicine was well tolerated in all patients, with the most common side effect diarrhoea in 16.3%. None of the patients had stopped colchicine treatment due to drug intolerance, nor showed colchicine resistance. Anakinra (human interleukin 1 receptor antagonist protein) was used in 1 female patient who had renal amyloidosis at age of 13 years: she had homozygous M694V mutation, and unfortunately died after prolonged intensive care unit care due to septicaemia; being the only mortality case of our cohort. Nonsteroidal anti-inflammatory drugs (NSAIDs) were used in FMF arthritis and during attacks. Patients who developed autoimmune diseases were treated accordingly as such: JIA patients needed Methotrexate; one received Adalimumab; Azathioprine was given to patients with Crohn’s Disease; one of them received Infliximab.

**TABLE 3.** Most frequent MEFV mutations among 196 Jordanian FMF children.

**Mutation**	**heterozygous**	**homozygous**	**Compound**	**Frequency (%)**
**M694V**	34	32	32	98 (50)
**V726A**	10	7	25	42 (21.4)
**E148Q**	21	1	16	38 (19.4)
**M680I**	10	7	14	31 (15.8)
**M694I**	3	1	15	19 (9.7)

**TABLE 4.** Most frequent MEFV mutations among 196 Jordanian FMF children.

**Mutation**	**heterozygous**	**homozygous**	**Compound**	**frequency**
M694V	34	32	32	98
V726A	10	7	25	42
E148Q	21	1	16	38
M680I	10	7	14	31
M694I	3	1	15	19

## DISCUSSION

Children below the age of 14 years were the only contributors of this cohort population, as patients who are older than 14 were being followed at adult clinic. The mean age was 7.8 years; comparable to reports from Egypt and Syria,^6,7^ while lower than what had been reported in a larger cohort in Turkish children.^8^ Although many reports of paediatric FMF showed female predominance as our report showed,^7,8,9^ Salah El-Din et al. reported male:female ratio of 1.3:1 in a cohort of 70 children, and found similar reports in Egypt and the United States; however, all were small cohorts.^6^ On the other hand, other paediatric and adult reports of different ethnic groups showed that FMF affects both genders similarly, and there was no statistical significance of gender predominance.^10^ Family history was positive for FMF in 29.1%, which is higher than what had been reported in the Iranian FMF registry,^11^ but lower than what was reported in Turkish children by Barut et al. (53.3%).^8^ Authors had explained higher rate of family history in their study population by the higher rate of consanguinity compared to general population. The majority of our patients had recurrent episodes of abdominal pain and/or fever, while about 40% of the cohort showed musculoskeletal symptom*s*, i.e. myalgia, arthralgia and arthritis, which is comparable to the multicentre international Eurofever registry for FMF in children.^12^ A larger cohort of 476 children from Jordan had been published by Majeed et al. in 1999,^13^ that showed a different clinical profile from what was previously reported in different ethnic groups, and from our cohort in regards to lower rates of abdominal pain, arthralgia, myalgia, and episodic fevers, but higher rates of arthritis and chest pain: Majeed and colleagues included any child with unexplained abdominal pain, and/or arthritis in their cohort without testing for MEFV gene, and this might have overlooked other differential diagnosis of FMF. We reported FMF arthritis only in 8.2% and chronic inflammatory arthritis in 3.1%, which is much lower than what had been reported in previous studies.^6,8,11,12^ The Nationwide Multicentre Study by the Turkish FMF Study Group^14^ had compared clinical phenotype of different ethnic groups; Turks, Jews, Arabs, and Armenians, and found that arthritis was much less reported among Arabs compared to Jews and Turks. The authors concluded that paediatricians practicing in the eastern Mediterranean counties still misdiagnosed attacks of arthritis, which might be the sole FMF presentation as acute rheumatic fever. This conclusion can explain why we never received a patient with episodic arthritis alone as suspected FMF. Vasculitis, especially Henoch-Schönlein purpura (HSP), polyarteritis nodosa (PAN), protracted febrile myalgia (PFM) and Behcet’s disease (BD) can either precede the diagnosis of FMF, or occur in the course of a previously known FMF.^15^ We had reported recurrent HSP, systemic leukocytoclastic vasculitis and recurrent oral ulcers which might evolve to BD in future. Other FMF-associated diseases were presented in our study, such as JIA, inflammatory bowel disease, and glomerulonephritis, but we did not report Systemic Lupus Erythematous, spondyloarthropathy, or isolated uveitis as was reported by the Nationwide Multi-centre Study by the Turkish FMF Study Group.^14^

Although renal amyloidosis is the most prevalent and still substantial problem, especially among Turks,^14^ it is not the only renal involvement: Hüzmeli and colleagues had studied 950 FMF patients who were referred to nephrology service for chronic proteinuria. They reported 9 patients with non-amyloid, IgA nephropathy, mesangioproliferative glomerulonephritis, membranous glomerulonephritis and immune complex glomerulonephritis.^16^ Similarly, we reported 1 case of renal amyloidosis (0.5%), which is similar to what had been reported in a paediatric cohort in Turkish children,^8^ and two cases with non-amyloid renal involvement, focal segmental glomerulosclerosis and minimal change nephrotic syndrome.

All patients in this study had been tested for 12 MEFV gene mutations, of which heterozygous were the most common in 44.4%, followed by compound heterozygous in 28.1%, and homozygous in 24%. This order is similar to what other paediatric studies had reported. However, what was remarkable in our cohort was the higher rate of mutation detection (96.4%); even more than reports from Egypt, Syria and Turkey (85.7%, 71.8%, 93.6% respectively).^6,7,8^

The five founder mutations were the most frequent detected mutations; M694V, V726A, E148Q, M680I, and M694I as shown in *Table 3*. A large cohort from Jordan published in 2015 studied the genetic profile of 1491 adult and paediatric patients who were clinically diagnosed as FMF; M694V, E148Q, V726A, M6801G/C, and M6941 were the most common detected mutations in the study population,^17^ which represents the same mutations frequency in our cohort.

Giaglis et al. had compared the frequency of the five most common MEFV mutations of their study group of the Greek population with different ethnic groups from other populations of the Mediterranean Basin. The M694V mutation was in the first order among Jews, Armenians, Arabs, Turks, Lebanese, Jordanian, Italians, and Greeks, but it was not in Cypriots and French. What was remarkable in our cohort was the higher rate of E148Q mutation (19.3%) in comparison with other reports.^18^ Out of five founder mutations, four reside on exon 10, while E148Q reside on exon 2, which has the highest carrier rate in different ethnic groups, and many studies demonstrated that E148Q is not likely to be a disease-causing mutation.^19^ However, Beheshtian et al. had compared mutations frequency in healthy individuals and FMF patients in different ethnic groups that included Turks, Arabs, Armenians, Jews, and Iranians, and found that E148Q detected in FMF patients ranged 1.8–32.7%, which supported the role of allelic variability in FMF expression.^20^

All our patients were treated with colchicine in a dose ranging from 0.5 mg to 2 mg, follow up of the patients according to the European League Against Rheumatism (EULAR) recommendations guidelines,^21^ treatment response was monitored by Auto-Inflammatory Diseases Activity Index (AIDAI) patient diary.^22^ Fortunately, all patients showed well-tolerated, either partial or complete colchicine response. We reported no cases of colchicine resistance based on the Ozen et al. (2017) definition which was adopted by EULAR; one or more attacks per month in compliant patients receiving the maximally tolerated dose for 6 months and more accounted up to 5%.^23^ We are not aware of why our patient had no colchicine resistance; it could be either due to higher rates of compliance and tolerability, or genetics.

## CONCLUSION

The five FMF founder mutations, M694V, V726A, E148Q, M680I, M694I are still the most common in Jordanian children. More awareness is needed in paediatricians for FMF presenting features, specially arthritis, and recurrent Henoch-Schönlein purpura. Colchicine treatment adherence is crucial for disease control and amyloidosis prevention, and non-compliance might be interpreted as colchicine resistance, which could lead to switching to unnecessary expensive drugs.
